# Depicting the genetic architecture of pediatric cancers through an integrative gene network approach

**DOI:** 10.1038/s41598-020-58179-0

**Published:** 2020-01-27

**Authors:** Clara Savary, Artem Kim, Alexandra Lespagnol, Virginie Gandemer, Isabelle Pellier, Charlotte Andrieu, Gilles Pagès, Marie-Dominique Galibert, Yuna Blum, Marie de Tayrac

**Affiliations:** 1 Univ Rennes, CNRS, IGDR (Institut de génétique et développement de Rennes) - UMR 6290, Rennes, France; 2 Somatic Cancer Genetics Department, Pontchaillou University Hospital, Rennes, France; 3 Pediatric Oncology Department, Pontchaillou University Hospital, Rennes, France; 4 Pediatric Immuno-Hemato-Oncology Unit, Angers University Hospital, Angers, France; 5 Molecular Genetics and Genomics Department, Pontchaillou University Hospital, Rennes, France; 6 Chemistry Oncogenesis Stress Signaling (COSS) Laboratory – INSERM U1242, Centre de Lutte Contre le Cancer (CLCC) Eugène Marquis, Rennes, France; 7 University Côte d’Azur, IRCAN (Institute for Research on Cancer and Aging of Nice) - CNRS UMR 7284 and INSERM U1081, Centre Antoine Lacassagne, Nice, France; 8 Biomedical Department, Centre Scientifique de Monaco, Monaco, Principality of Monaco; 9 Programme Cartes d’Identité des Tumeurs (CIT), Ligue Nationale Contre le Cancer, Paris, France

**Keywords:** Cancer genomics, Gene expression, Paediatric cancer, Computational science

## Abstract

The genetic etiology of childhood cancers still remains largely unknown. It is therefore essential to develop novel strategies to unravel the spectrum of pediatric cancer genes. Statistical network modeling techniques have emerged as powerful methodologies for enabling the inference of gene-disease relationship and have been performed on adult but not pediatric cancers. We performed a deep multi-layer understanding of pan-cancer transcriptome data selected from the Treehouse Childhood Cancer Initiative through a co-expression network analysis. We identified six modules strongly associated with pediatric tumor histotypes that were functionally linked to developmental processes. Topological analyses highlighted that pediatric cancer predisposition genes and potential therapeutic targets were central regulators of cancer-histotype specific modules. A module was related to multiple pediatric malignancies with functions involved in DNA repair and cell cycle regulation. This canonical oncogenic module gathered most of the childhood cancer predisposition genes and clinically actionable genes. In pediatric acute leukemias, the driver genes were co-expressed in a module related to epigenetic and post-transcriptional processes, suggesting a critical role of these pathways in the progression of hematologic malignancies. This integrative pan-cancer study provides a thorough characterization of pediatric tumor-associated modules and paves the way for investigating novel candidate genes involved in childhood tumorigenesis.

## Introduction

Cancer remains the leading cause of death by disease in children of less than fourteen years of age^1^. Improving the management of pediatric cancer is essential and will benefit from more accurate diagnosis, new personalized treatment and development of specific and less damaging therapies. To face these challenges, it is necessary to unravel the complete genetic repertoire of pediatric malignancies. Recent studies have improved the understanding of the genetics of childhood cancer, but have mainly focused on depicting the germline and somatic mutational landscape of these diseases^2–4^.

Several evidences demonstrated that the biology and genetics of pediatric cancers set them apart from adult tumors^4,5^. Childhood cancers have a 14-times lower mutation rate compared to adult tumors and mostly arise from mutations in few driver genes. Somatic alterations mostly target a handful of major genes such as *CDKN2A, NOTCH1, NRAS, KRAS* or *TP53*, and pathways disrupted by driver alterations are either common to cancer (e.g. cell cycle) or specific to pediatric cancer histotypes^4^. More than half of the driver genes are restricted to one cancer histotype and 83% of them are not shared between hematologic and solid tumors. This indicates that certain genes and pathways are exclusively dysregulated in a single type of childhood cancer.

Regarding hereditary predisposition, genome-wide studies reported that pathogenic germline variants were identified in 8–10% of the affected children and adolescents^2,6–8^. This proportion is likely underestimated considering that only cancer-related genes were analyzed for pathogenicity in these studies. To date, over 100 cancer predisposition genes have been described and most of the associated pathogenic germline variants were loss of function mutations in DNA or double-stranded break repair genes^2,3,8^. The total spectrum of cancer-predisposition genes involved in childhood tumorigenesis still remains to be uncovered.

Tumor initiation and progression result from complex interplay between germline and somatic events that shape the transcriptional landscape of tumors^9,10^. Integration of transcriptome-based knowledge has emerged as a powerful method for prioritizing genomic alterations in cancers^11^. Statistical network modeling is essential for interpreting genotype-to-phenotype relationships or discerning transcriptional regulatory programs^12–14^. Studies reported that mature pediatric tumors mirror the conserved transcriptional programs of embryonic cell populations that have been subject to genomic changes^15^. A system-level understanding of how the genetic mutations affect transcriptional profile has been provided in adult pan-cancer data^16^. Such analyses revealed common functional gene clusters that are shared by multiple adult cancer types.

In onco-pediatric research, construction of co-expression networks achieved interesting results in identifying predictive molecular biomarkers and in unraveling differential regulatory molecular programs by analyzing matched normal-tumor samples^14,17^. The published studies have only focused on deciphering co-expression networks of one particular histotype and, therefore, lack to provide a global view of both common and histotype-specific processes that drive childhood tumorigenesis. This requires a deep exploration of the co-expression network obtained by analyzing pan-cancer childhood data.

Here, we carried out computational analyses of the transcriptome data of 820 pediatric cancer samples selected from the Treehouse Childhood Cancer Initiative (TCCI) dataset across six cancer histotypes. We constructed a co-expression network using weighted gene co-expression network analysis (WGCNA) to capture transcriptional relationships between genes in pediatric cancers. We associated the resulting modules with tumor types by examining their transcriptional profiles and by characterizing their biological functions. We determined the most connected genes within modules and highlighted their biological relevance to different tumor types. We investigated for the over-representation of pediatric cancer gene sets in these modules and mapped them into the co-expression network. Our integrative analysis provides a working frame for investigating candidate genes involved in pediatric tumorigenesis through the deep-level exploration of modules specifically associated with childhood cancers.

## Results

### Childhood cancer histotypes are characterized by distinct transcriptional profiles

**Figure 1:**
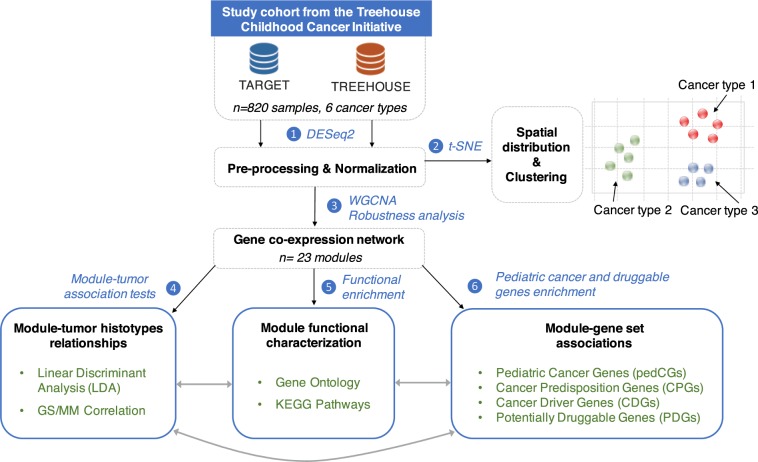
Workflow of the overall integrative approach to decipher the modules associated with pediatric tumors. General workflow of the pan-cancer integrative study that consisted in the selection of 820 pediatric cancer samples from the Treehouse Childhood Cancer Initiative, followed by a pre-processing and normalization procedure of the RNA-Seq data. We constructed a co-expression network and identified 23 co-expression modules of genes sharing similar expression profiles across pediatric cancer samples. We performed a deep multi-layer examination of the resulting modules to identify module-tumor relationships, enrichment in biological processes and in relevant pediatric cancer gene sets. WGCNA, Weighted Gene Co-Expression Network Analysis; t-SNE, t-distributed Stochastic Neighbor Embedding.

We hypothesized that transcriptome data from pediatric cancer samples could provide a thorough understanding of the key genes and pathways implicated in childhood tumorigenesis. We thus developed an integrative study for which the general workflow is displayed in Fig. 1.

**Figure 2:**
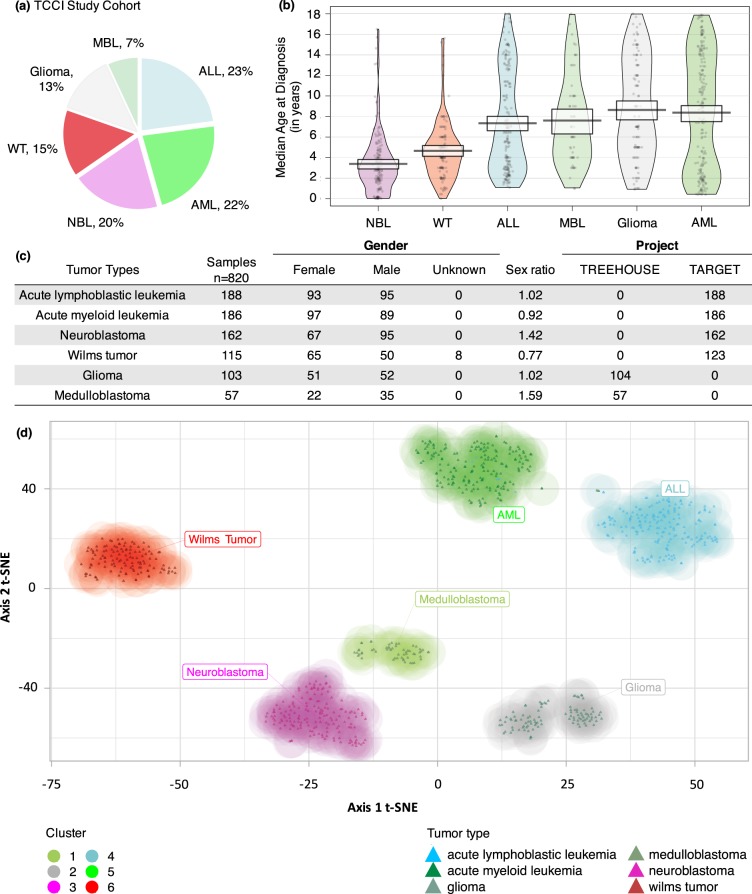
Clinical description of patient samples selected from the Treehouse Childhood Cancer Initiative (TCCI) dataset. **(a)** Distribution of cancer histotypes analyzed in our study that includes 820 tumor samples in patients with age at diagnosis younger than 18 years old selected from the TCCI dataset. **(b)** Distribution of patient ages at diagnosis (in years) by tumor types. **(c)** Clinical characteristics of patient samples in our study cohort by gender and project. **(d)** Distribution visualization of pediatric cancer samples using t-SNE analysis, a nonlinear multivariate method that embeds the high-dimensional data into a two-dimensional space. Each dot represents a patient sample, colored by tumor types. Hierarchical clustering of cancer samples is depicted by the 6 colored density maps and clusters are labeled according to the most represented cancer type. ALL, Acute Lymphoblastic Leukemia; AML, Acute Myeloid Leukemia; MBL, Medulloblastoma; NBL, Neuroblastoma; WT, Wilms Tumor.

We selected 820 childhood tumor samples across six different cancer types from the TCCI dataset (Fig. 2a; Additional File 2: Table S1). The median age at diagnosis (MAD) of the patients ranged from 3 to 9 years old depending on tumor types (Fig. 2b). The MAD was higher compared to previous studies for Neuroblastoma (NBL; 2.9 years old in our study vs 1.5 years old reported previously)^18^, Wilms Tumor (WT; 4 vs 3.5 years old)^19^, Medulloblastoma (MBL; 7 vs 6 years old)^20^ and Acute Myeloid Leukemia (AML; 8.8 vs 6.4 years old)^21^. The median age was consistent with recent reports for Acute Lymphoblastic Leukemia (ALL; 6.4 vs 6.5 years old)^22^, but lower for glioma with 8 vs 9 years old^23^. Consistent with the American Cancer Society statistics of 2014, we found a higher incidence of males in NBL (sex ratio = 1.42) and MBL (sex ratio = 1.59), along with a slight female preponderance in WT (sex ratio = 0.77) (Fig. 2c).

We next applied a t-distributed stochastic neighbor embedding (t-SNE) algorithm on the RNA-Seq data to refine groups of childhood tumors by projecting the patient samples in a low-dimensional space based on their transcriptional features. Hierarchical clustering of the resulting coordinates revealed six clusters matching the pediatric tumor histotypes (Fig. 2d; Additional file: Table S1). We observed a clear segregation between hematologic and solid tumors supporting the notion that acute childhood leukemias have distinct transcriptional profiles as compared to solid tumors. Among childhood solid tumors, NBL, MBL and glioma shared more similar expression patterns than WT samples. Two subgroups were outlined in gliomas, with expression profiles representative of the PDGFRA-amplified vs PDGRA-non amplified gene signatures described in DIPG tumors (Data not shown)^24^. Considering the distinct embryonic origin of childhood tumors, our findings demonstrate that each pediatric cancer type has a specific transcriptome signature.

### Childhood cancer modules are representative of specific tumor histotypes

**Figure 3:**
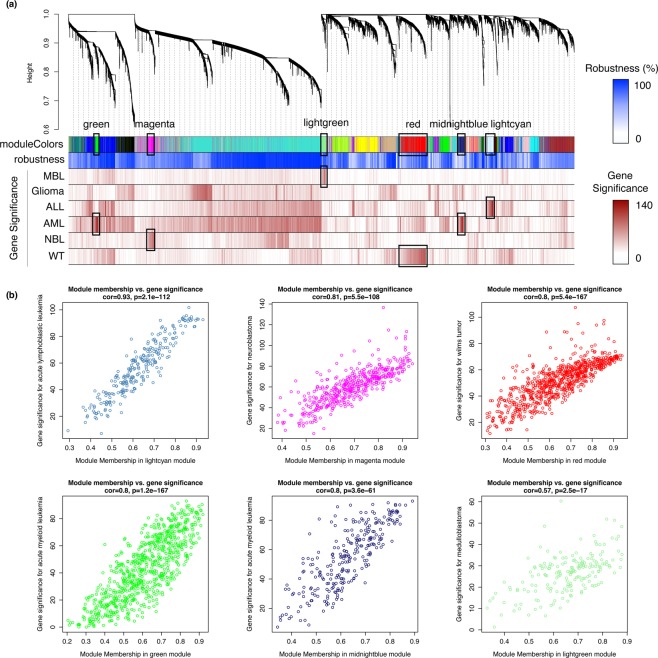
Identification of cancer-histotype specific modules that are associated with distinct pediatric tumors. **(a)** Dendrogram showing genes sharing similar expression profiles across pediatric tumor samples and gathered in modules identified by WGCNA. Each branch of the dendrogram represents a gene assigned to one of the 23 colored-label modules, the grey module gathers all non-assigned genes. Bars below are color-coded with a white (low percentage) to blue (high percentage) gradient to represent the robustness of the gene-module association; and a white (low value) to red (high value) gradient to give information on the absolute value of gene significance (GS; i.e., gene-tumor relationship) across tumor types. Six modules were highlighted (black box) due to high levels of GS for six distinct cancer histotypes. **(b)** Scatterplot representing the correlation between the absolute values of GS and Module Membership (MM; i.e., gene-module relationship) of the co-expressed genes in a cancer-histotype specific module. The correlation coefficient and statistical probability are displayed (on top) and dots are colored by module colored-label. ALL, Acute Lymphoblastic Leukemia; AML, Acute Myeloid Leukemia; MBL, Medulloblastoma; NBL, Neuroblastoma; WT, Wilms Tumor.

After demonstrating that pediatric tumors were characterized by specific gene expression profiles, we have undertaken a network-based approach to identify modules of genes particularly associated with childhood tumors. We constructed modules of genes sharing highly similar expression patterns across pediatric pan-cancer samples by performing WGCNA analysis on the transcriptome data of the study cohort. We identified 23 co-expression modules, labeled by color (Fig. 3a). For all genes, we assessed their biological relevance with regard to different tumor types using the gene significance (GS) measure and all the results have been reported (Additional File 1: Table S2). In six modules, the co-expressed genes exhibited high values of GS and high specificity of associations with histologic tumor subtypes (Fig. 3a). The stability and reliability of the identified modules were validated by bootstrapping and robustness analyses (Additional File 1: Table S3; Additional File 2: Fig. S1).

Cancer-histotype specific modules were defined as modules exhibiting highly specific association with a particular cancer type on the basis of (1) their gene expression levels in the tumor samples and (2) their gene significance levels towards the cancer histotype. We first assessed module-tumor relationships by using Linear Discriminant Analysis (LDA) approach that maximizes the separation between tumor types based on the expression profiles of the modules (Additional File 1: Table S3; Additional File 2: Fig. S2a,b). A module was found strongly associated with a childhood cancer, when its absolute average expression levels in tumor samples was higher than an empirical threshold of 0.06 (see Methods). We identified six module/tumor associations, such as lightgreen/MBL (mean = 0.11), red/WT (mean = 0.08), magenta/NBL (mean = 0.06) and lightcyan/ALL (mean = 0.06). The genes of the midnightblue module were under-expressed in AML samples as compared to other cancers (mean = −0.06), as the genes of the tan module in glioma samples (mean = −0.06). Nine modules showed exclusive transcriptional patterns between cancer solid (CSTs) and liquid (CLTs) tumors. The black, blue, brown and darkred modules demonstrated high expression in CLTs vs low expression in CSTs, whereas the turquoise, pink, midnightblue, yellow and darkturquoise modules had high expression in CSTs vs low expression in CLTs. Transcriptional profiles of the magenta, red, lightgreen, lightcyan, green and midnightblue module exhibited strong specificity towards NBL, WT, MBL, ALL, AML and AML, respectively (Additional File 2: Fig. S2c). We then performed statistical analyses that revealed strong positive correlations between the module membership (MM) of a gene and its biological significance towards a particular tumor histotype. These findings supported module/tumor associations such as lightcyan/ALL (R^2^ = 0.93, *p* < 0.001), magenta/NBL (R^2^ = 0.81, *p* < 0.001), red/WT (R^2^ = 0.8), midnightblue/AML (R^2^ = 0.8, *p* < 0.001), green/AML (R^2^ = 0.8, *p* < 0.001) and lightgreen/MBL (R^2^ = 0.51, *p* < 0.001) (Fig. 3b). A low correlation was identified between the tan module and glioma (R^2^ = 0.39, *p* < 0.001). To further demonstrate the significance of these module/tumor associations, we found that the second highest levels of correlation decreased to levels less than 0.5 with the other cancer types: lightcyan/WT (R^2^ = 0.22, *p* < 0.001), magenta/AML (R^2^ = 0.38, *p* < 0.001), red/AML (R^2^ = 0.48, *p* < 0.001), green/MBL (R^2^ = 0.45, *p* < 0.001), midnightblue/glioma (R^2^ = 0.35, *p* < 0.001), lightgreen/AML (R^2^ = 0.27, *p* < 0.001) (Additional File 2: Fig. S2d). The modules that fulfilled both established criteria and were defined as cancer-histotype specific and named according to their associated tumor, as the magenta-NBL, red-WT, lightcyan-ALL, lightgreen-MBL, midnightblue-AML and green-AML modules.

### Cancer-histotype specific modules gather cornerstone biological functions involved in the physiopathology of the associated tumor

Considering the associations between the modules and the specific tumor types, we reasoned that exploring the biological functions of the genes within modules would shade light on the subtype-specific processes implicated in pediatric cancers. To investigate this, we performed functional enrichment analyses using GO and KEGG annotation terms for each module (Additional File 1: Table S4).

**Figure 4:**
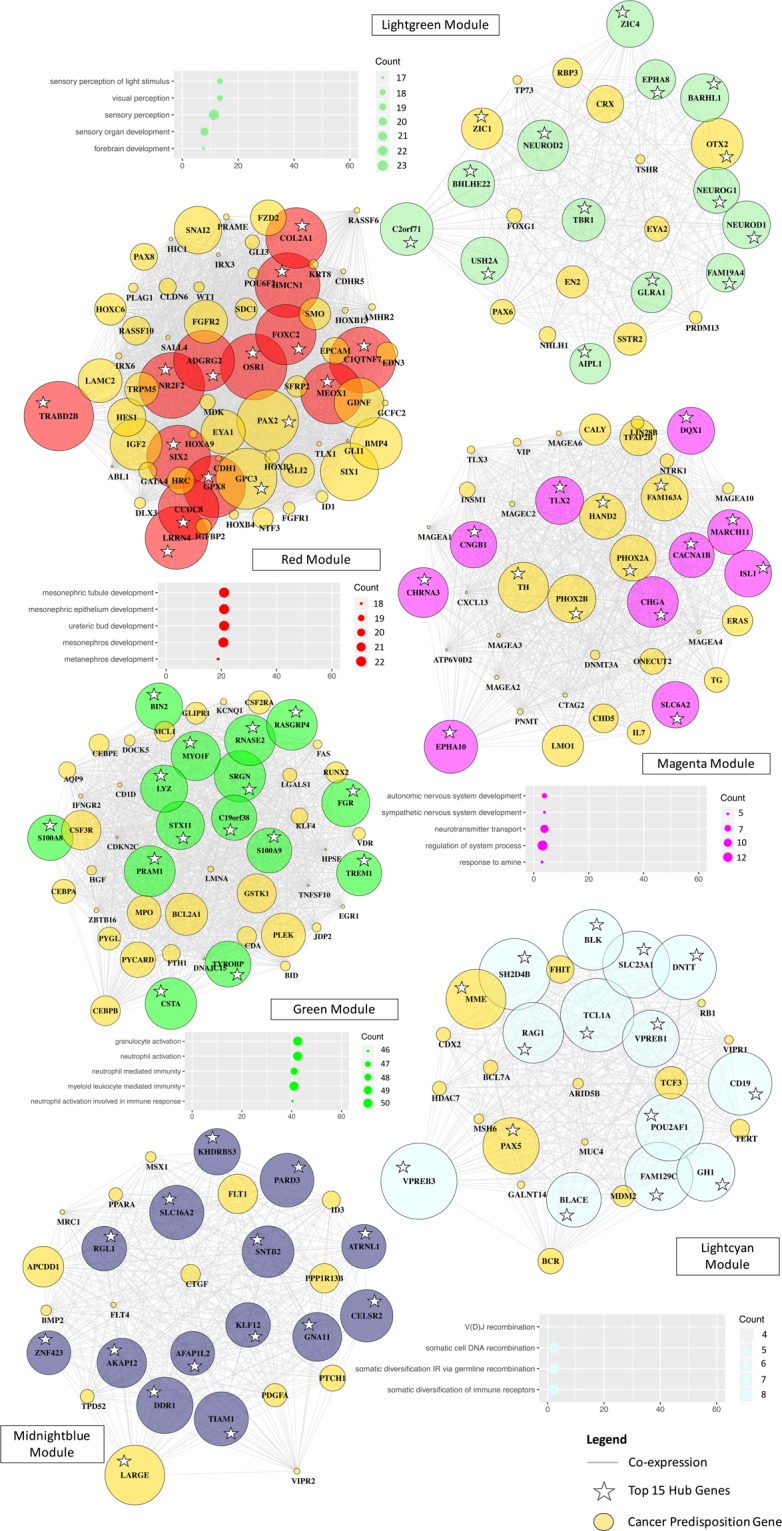
Hub genes and biological processes of the childhood cancer-histotype specific modules. Visualization of the network plots for the magenta, red, lightgreen, lightcyan, midnightblue and green modules using igraph R library. Genes are represented as nodes and edges as the connectivity between gene pairs derived from the TOM. The size of the node is proportional to the sum of connections of the gene within the module. Nodes are labeled according to the HGNC symbols. For each module, the top 15 hub genes are highlighted by a white star and the cancer predispostion genes by gold-colored nodes. Biological characterization of the cancer-histotype specific modules is displayed as scatterplots below the associated network plot, except for the midnightblue module that did not show any significant enrichment. These scatterplots show the top 5 enriched canonical pathways in Gene Ontology (GO) annotation terms (on the left). Statistical probabilities are adjusted for multiple comparisons (FDR < 0.01) and reported as –log10(FDR). Dots are colored by module colored-label (on the right) and sized by the count number of genes matching the biological process in the module. IR, Immune response.

We found that genes co-expressed in the magenta-NBL module were involved in the development of the autonomic (Fold Change (FC) = 22; FDR < 0.001) and sympathetic nervous system (FC = 36; FDR < 0.001), in line with the physiopathology of NBL that derives from postganglionic sympathetic neuroblasts (Fig. 4). The red-WT module was enriched in genes taking part in metanephros (FC = 29; FDR < 0.001), mesonephros (FC = 28; FDR < 0.001) and ureteric bud development (FC = 29; FDR < 0.001), which was consistent with the tumor initiation mechanisms of WT. Indeed, this embryonic tumor develops from residual ureteric bud and metanephric mesenchyme/blastema^25^. The lightcyan-ALL module was enriched in genes related to the antigen recognition response by somatic gene rearrangement with V(D)J recombination (FC = 44; FDR < 0.001). Abnormal recombination during somatic rearrangements of surface immunoglobulin (Ig) and T cell antigen receptor (TCR) genes has been described in the transformation of lymphoid cells^26^. The lightgreen-MBL module was functionally related to visual (FC = 16; FDR < 0.001) and sensory perception (FC = 8; FDR < 0.001), along with forebrain development (FC = 7; FDR < 0.001). This is in accordance with the aberrant differentiation of the most aggressive subgroup of MBL in the photoreceptor program^27^. The over-expressed genes in the green-AML module were linked to myeloid-mediated immunity processes (FC = 14; FDR < 0.001). No enrichment was found in either GO or KEGG annotation terms for the midnightblue-AML module. Taken collectively, five out of the six cancer-histotype specific modules were enriched in biological functions consistent with the tissue origins and physiopathology of the related childhood cancer. The modules associated with pediatric tumors encompass genes implicated in developmental processes, supporting the close link between organogenesis and tumorigenesis in childhood cancers.

### Co-expression modules functionally related to canonical oncogenic and onco-hematologic pathways in childhood cancers

**Figure 5:**
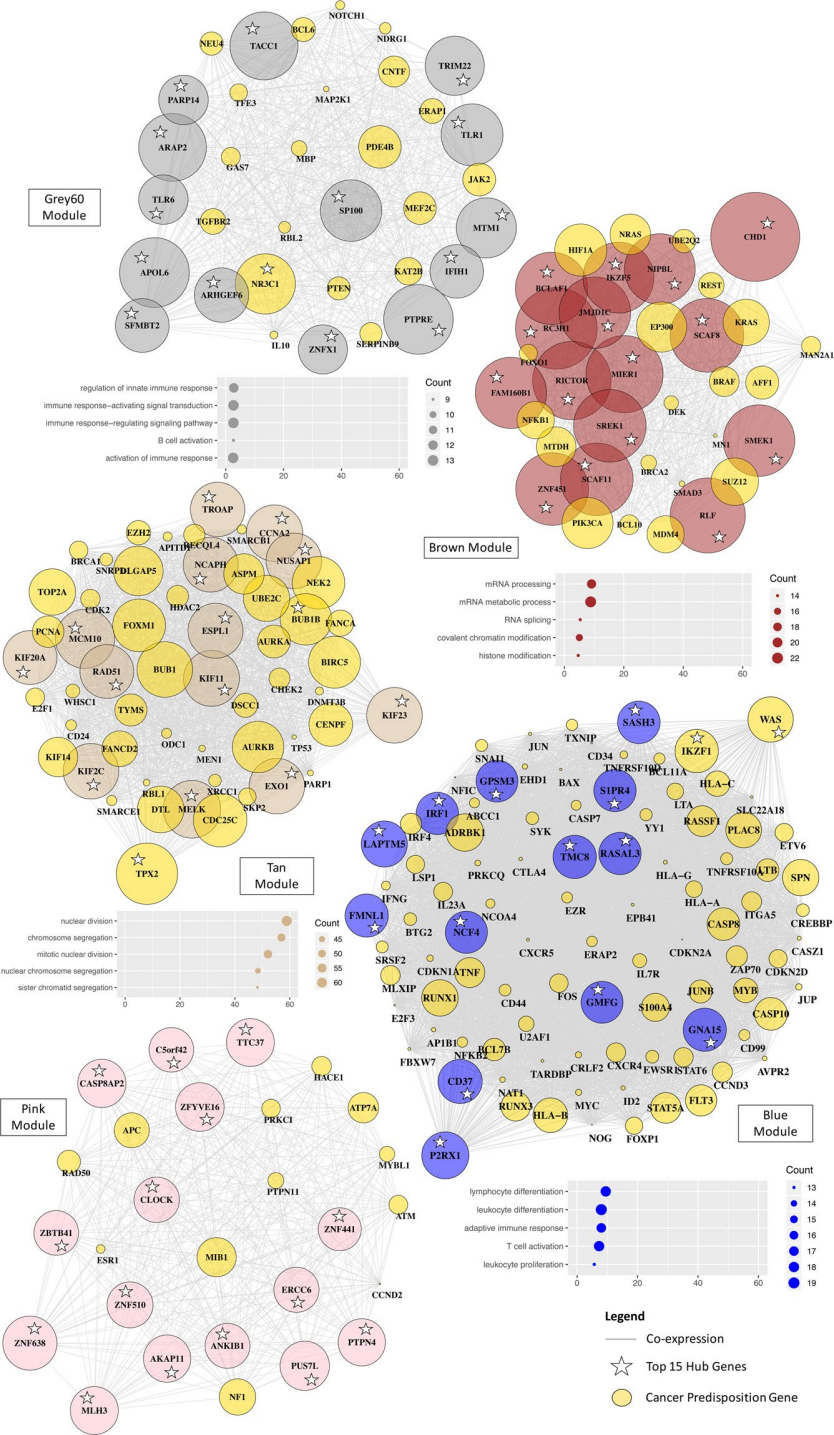
Hub genes and biological processes of the oncogenic and onco-hematologic modules in childhood cancers. Visualization of the network plots for the grey60, brown, tan, blue and pink modules using igraph R library. Genes are represented as nodes and edges as connectivity between gene pairs derived from the TOM. The size of the node is proportional to the sum of the connections of a gene within the module. Nodes are labeled according to the HGNC symbols. For each module, the top 15 hub genes are highlighted by a white star and the cancer predisposition genes by gold-colored nodes. Biological characterization of the cancer-histotype specific modules is displayed as scatterplots below the associated network plot, except for the pink module that did not show any significant enrichment. These scatterplots show the top 5 enriched canonical pathways in Gene Ontology (GO) annotation terms (on the left). Statistical probabilities are adjusted for multiple comparisons (FDR < 0.01) and reported as –log10(FDR). Dots are colored by module colored-label (on the right) and sized by the count number of genes matching the biological process in the module.

Functional examination of the 17 non-cancer-histotype specific modules revealed significant associations with either canonical oncogenic or onco-hematologic processes (Fig. 5; Additional File 1: Table S4). The tan module was enriched in cell cycle processes such as nuclear division (FC = 16; FDR < 0.001), but also DNA repair (FC = 9; FDR < 0.001) and replication (FC = 13; FDR < 0.001) that are commonly disrupted by pathogenic germline variants in childhood cancers^2,8^. The blue module was involved in lymphocyte differentiation (FC = 10; FDR < 0.001) and proliferation (FC = 8; FDR < 0.001) which is consistent with the pathogenesis of ALL. Likewise, the grey60 module was related to the B-cell ALL subtype with functional enrichment in B cell activation (FC = 7; FDR < 0.001) and differentiation (FC = 10; FDR < 0.001). Despite functions specific to the B-cell ALL subtype, the grey60 module showed higher levels of expression in both acute leukemias and glioma samples, thereby, not fulfilling the « histotype-specific » criteria. The brown module was over-represented in genes linked to post-transcriptional and epigenetic processes, such as mRNA processing (FC = 9; FDR < 0.001) and histone modification (FC = 6; FDR < 0.001), which are believed to contribute to relapse in acute leukemias^28^. Additional modules were associated with biological mechanisms having known implications in pediatric tumorigenesis, see Additional File 1: Table S4.

### Pediatric cancer genes are significantly enriched in the associated cancer-histotype specific module

**Figure 6:**
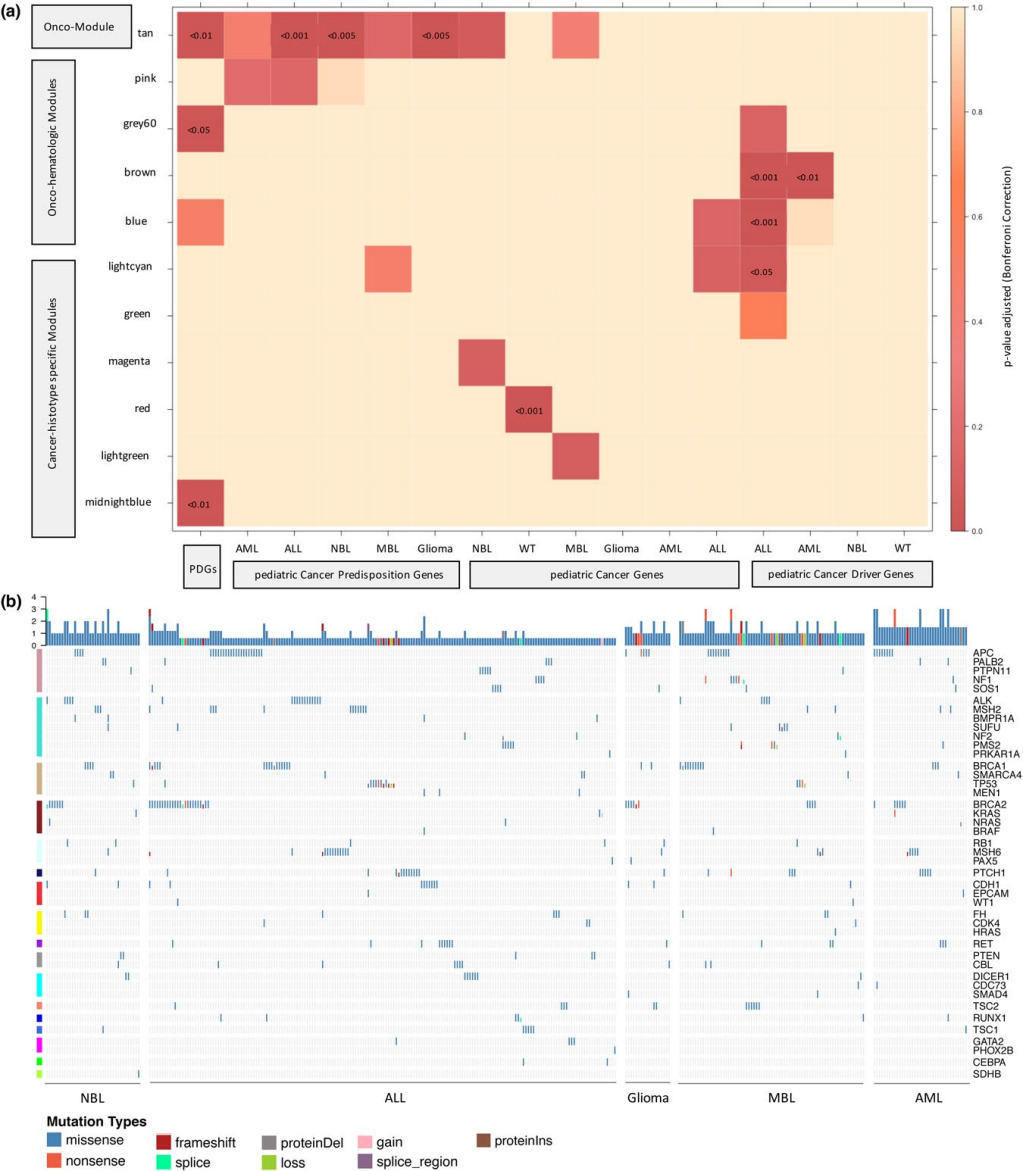
Enrichment analysis and mapping of literature-based pediatric cancer gene sets in childhood cancer modules. **(a)** Enrichment in relevant pediatric cancer gene lists for childhood cancer modules. Heatmap using OncoPrint displaying the over-representation results for Potentially Druggable Genes (PDGs), Cancer Predisposition Genes (CPGs), pediatric Cancer Predisposition Genes (pedCPGs) and pediatric Cancer Driver Genes (pedCDGs) associated with pediatric cancer cancers gene lists in oncogenic (tan), onco-hematologic (pink, grey60, brown, blue) and cancer-histotype specific (lightcyan, green, magenta, red, lightgreen, midnightblue) modules. Cells are color-coded according to the adjusted p-values using Bonferroni correction (legend on the right) and values are shown when p < 0.05. **(b)** Landscape of germline alterations in cancer genes across co-expression modules and pediatric tumor samples. Distribution of publicly available germline mutations in 43 autosomal dominant predisposing cancer genes identified by Zhang and colleagues (2015). Color-coded alterations (legend on the bottom) are displayed in cells with their frequency (barplots on top) for each sample (in columns). Samples are split in different grids by tumor types. Genes (in rows) are sorted by mutational rates and separated by label-colored modules in the following order: pink, turquoise, tan, brown, lightcyan, midnightblue, red, yellow, purple, grey60, cyan, salmon, blue, royalblue, magenta, green and greenyellow. ALL, Acute Lymphoblastic Leukemia; AML, Acute Myeloid Leukemia; MBL, Medulloblastoma; NBL, Neuroblastoma; WT, Wilms Tumor.

Based on the PediCan database^29^, we defined different lists of pediatric cancer genes (pedCGs) implicated in ALL (113 genes), AML (32 genes), WT (49 genes), MBL (113 genes), NBL (166 genes) and glioma (22 genes) (Additional File 1: Table S2). We then tested their enrichment for each of the 23 co-expression modules and all the results are available in the Additional File 1: Table S5. We found the magenta-NBL module as significantly enriched in NBL pedCGs (OR = 2.9 [1.5–5.1]; *p* < 0.001), the lightcyan-ALL module in ALL pedCGs (OR = 4.4 [1.8–9.2]; *p* < 0.001), the red-WT module in WT pedCGs (OR = 6.9 [3.3–13.4]; *p* < 0.001), and the lightgreen-MBL module in MBL pedCGs (OR = 5.4 [2.1–11.7]; *p* < 0.001) (Fig. 6a; Additional File 1: Table S5).

Overall, 4 out of the 6 cancer-histotype specific modules were significantly enriched in the pediatric cancer genes of the associated tumor type. These findings support that genes in these cancer-histotype specific modules should be given higher priority in variants prioritization methodologies applied to childhood cancers.

### Pediatric cancer predisposition genes and driver genes are enriched in childhood cancer modules

Recently, Zhang and colleagues^2^ depicted the germline mutational landscape of pediatric tumors through a comprehensive pan-cancer study in a large cohort of children and adolescents. The pediatric cancer predisposition genes (pedCPGs) were defined as cancer-related genes harboring pathogenic germline mutations in childhood cancer patients. We mapped the literature-based pedCPGs into the constructed childhood cancer co-expression network (Fig. 6b). We observed that cancer-related genes were not exclusively mutated in one histotype but rather altered in multiple pediatric tumors. For each module, we performed gene enrichment analyses in pedCPGs of the studied tumor types. We also tested for over-representation in potentially druggable genes (PDGs, i.e. genes having a direct or indirect targeted treatment available or under development) to assess the status of druggability of the modules.

This analysis revealed that the tan (PDGs, OR = 3.2 [1.65–5.75]; *p* < 0.001) and midnightblue-AML (PDGs, OR = 3.94 [1.9–7.36]; *p* < 0.001) modules encompassed most of the clinically actionable genes. We found the oncogenic (tan) module to be significantly enriched in pediatric cancer predisposition genes for 4 out of the 5 tested histotypes (NBL pedCPGs, OR = 9.0 [3.3–21.2]; *p* < 0.001; ALL pedCPGs, OR = 8.3 [3.9–16.3]; *p* < 0.001; MBL pedCPGs, OR = 9.9 [2.4–31.3]; *p = *1.4 × 10^–3^; glioma pedCPGs, OR = 9.7 [3.5–23]; *p* < 0.001) (Fig. 6a). No data were available for WT, as this tumor has not been studied by Zhang and colleagues^2^ (Fig. 6a; Additional File 1: Table S5). Most of the germline alterations affecting cancer genes in pediatric acute leukemia were significantly enriched in the pink module (ALL pedCPGs, OR = 8.2 [1.7–8.9]; *p* = 1.3 × 10^–3^; AML pedCPGs, OR = 6.9 [2.0–19.0]; *p* = 1.6 × 10^–3^). These results suggest that genes in the tan module, when mutated, are likely contributing to tumor initiation in multiple childhood cancers. We also identified a novel onco-hematologic module (pink) that gathers genes believed to be early genetic determinants in pediatric acute leukemia (Fig. 5).

Ma and colleagues^4^ identified pediatric cancer driver genes (pedCDGs) in a large cohort of childhood cancer patients. We recovered the published data of this study to build lists of cancer driver genes significantly mutated in ALL (106 genes), AML (33 genes), WT (12 genes) and NBL (8 genes) (Additional File 1: Table S2). The brown module was enriched in ALL pedCDGs (OR = 4.1 [2.4–6.7]; *p* < 0.001) and AML pedCDGs (OR = 6.1 [2.5–13.7]; *p* < 0.001). The ALL driver genes were over-represented in the grey60 (OR = 4.55 [1.8–9.9]; *p* = 1.4 × 10^–3^) and lightcyan-ALL (OR = 4.7 [2.0–9.9]; *p* < 0.001) modules. We demonstrated that genes frequently altered by somatic alterations in pediatric ALL were significantly enriched in the grey60 module related to B cell development and in the lightcyan-ALL module. The grey60 module could therefore, play a key role in the ALL tumorigenesis and was also associated with a favorable status of druggability (PDGs, OR = 3.71 [1.64–7.35]; *p* = 1.2 × 10^−3^).

### Pediatric cancer genes are enriched in the hub genes of childhood cancer modules

In network-based approach, hub genes are often identified as key regulators of the observed processes^14,16,17,30^, here the pathogenesis of childhood cancers. We defined hub genes as the most interconnected genes within a module. To provide novel insights into potential key regulators of childhood cancers, we performed an in-depth evaluation of their hub genes and focused on the top 15 hub genes (Fig. 4).

The paired like homeobox 2B (*PHOX2B*) gene is one of the major predisposition gene for NBL^31^ and was identified as a key regulator in the magenta-NBL module. Other hub genes of this module were shown as essential for neural differentiation of the sympathoadrenal lineage (*PHOX2A, HAND2, PHOX2B, ISL1*) and comprised a novel candidate gene for NBL (*ISL1*)^32,33^. The paired box 2 (*PAX2)* was among the hub genes of the red-WT module and believed to be a tumor-inducing gene in WT with key role in kidney cell differentiation^34^. The major gene of predisposition to Wilms Tumor (*WT1*) was, however, ranked 483^rd^, as its expression was high in both WT and AML samples (Additional File 2: Fig. S3). In the lightcyan-ALL module, we identified as a key regulator the paired box 5 gene (*PAX5*) gene, known as the major predisposition gene in B-cell ALL^35^, one of the direct target of PAX5 (*CD19*) and a key player in B-cell differentiation (*TCL1A*)^36^. In the lightgreen-MBL module, the hub genes were involved in neurogenesis, particularly in the forebrain (*OTX2, TBR1*) and cerebellar development (*OTX2, BARHL1*, *ZIC1* and *ZIC4*) with predominant expression in MBL^37–41^. Two of these hub genes (*OTX2, NEUROD1)* were the conductors of key transcriptional programs in the Group 3 subtype of MBL, the most aggressive subtype of MBL^37^. The hub genes in the green-AML module encoded protein with roles in leukemogenesis and myeloid differentiation (*PRAM1*, *RASGRP4*, *S100A9*) or subject to recurrent alterations in infant AML (*MYO1F*)^42–45^. Statistical analyses supported that pediatric cancer genes were enriched among the hub genes of the six cancer-histotype specific modules (OR = 1.9 [1.2–2.9], *p* = 0.004). The pediatric cancer genes of one tumor type were enriched among the hub genes of the associated module (WT/red, OR = 4.8 [1.8–14.4], FDR = 0.021; NBL/magenta, OR = 3.1 [1.5–6.4], FDR = 0.032; lightgreen/MBL, OR = 4.8 [1.7–12.1], FDR = 0.032; ALL/lightcyan, OR = 3.8 [1.4–9.2], FDR = 0.033).

Many of the key regulators in the canonical oncogenic (tan) module were involved in the processes leading to tumor cell proliferation and survival (*TPX2*, *NCAPH*, *KIF11*, *NUSAP1*, *KIF23*, *MCM10*) but most of them have not been associated with pediatric tumors (Fig. 5). Pediatric cancer predisposition genes for glioma (OR = 8 [2.7–21.6], FDR = 0.004), ALL (OR = 5.8 [2.6–12.2], FDR = 0.001) and NBL (OR = 6.5 [2.2–16.7], FDR = 0.008) were enriched among the hub genes of the tan module. The hub genes of the grey60 module had major roles in innate immune recognition and activation (*TLR1*, *TLR6, PTPRE*, *TRIM22*, *PARP14*, *ARHGEF6*, *IFIH1*, *NR3C1*) (Fig. 5). Considering that hematologic malignancies employ unique immune evasion strategies as compared to solid malignancies, the hub genes of the grey60 module could constitute promising innate immune targets^46^. The key regulators of the brown module were involved in chromatin and histone modifications (*CHD1*, *JMJD1C*, *NIPBL*) and RNA metabolic processes (*RC3H1*, *SREK1*, *PHRF1*, *BCLAF1*). Some of these hub genes were either considered essential to the survival of AML cells (*JMJD1C*) or identified as fusion partners (*CHD1*) of the major player in hematologic malignancies (*RUNX1)*. The hub genes of the blue module take part in immune response mechanisms (*IRP1*, *RASL3*, *FMNL1*) and comprised one critical regulator of lymphoid differentiation (*IKZF1*) that is frequently deleted or mutated in B-cell precursor ALL^47^. The cancer driver genes of ALL were enriched among the hub genes of the blue module (OR = 2.3 [1.5–3.7], FDR = 0.004).

## Discussion

**Figure 7:**
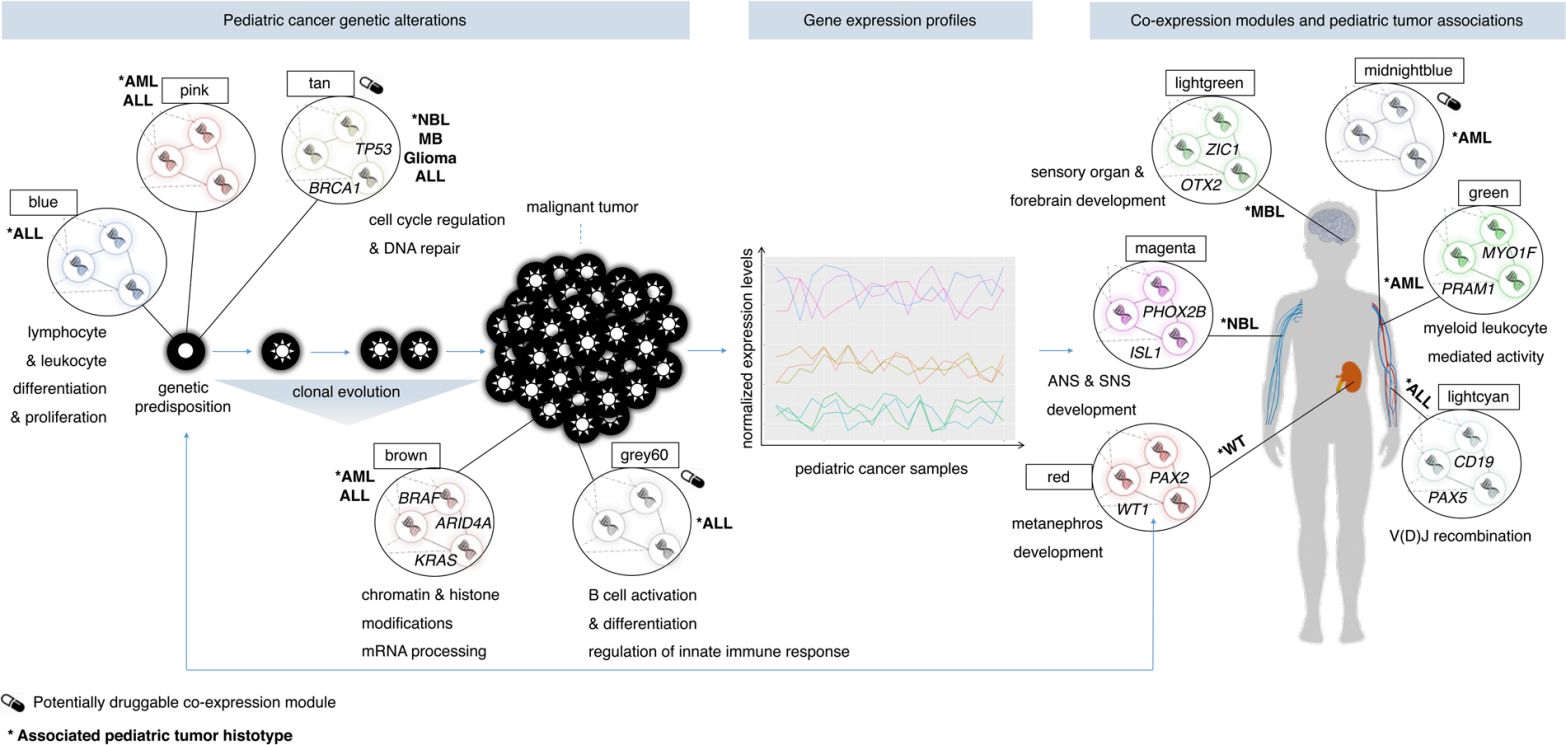
Transcriptome-based approach to identify co-expression modules associated with pediatric tumors. Deep multi-layer inspection of the pediatric co-expression network indicates that the canonical oncogenic (tan) module significantly regroups the pediatric cancer genes altered by germline mutations, likely contributing to tumor initiation of multiple pediatric tumors. The tan module is functionally involved in cell cycle regulation and DNA repair and enriched in genes subject to targeted therapies. In pediatric acute leukemia, cancer predisposition genes are enriched in the pink and blue modules, whereas cancer driver genes are over-represented in the brown and grey60 modules. The module-tumor association tests and functional enrichment analyses highlight processes exclusively dysregulated in specific childhood tumor histotype. These six cancer-histotype specific modules are linked to biological functions overlapping with the physiopathology of the associated tumor histotype. We also highlight that hub genes within these cancer-histotype specific modules are known pediatric cancer predisposition genes (e.g. *PHOX2B*, *PAX5*). ALL, Acute Lymphoblastic Leukemia; AML, Acute Myeloid Leukemia; ANS, Autonomic Nervous System; MBL, Medulloblastoma; NBL, Neuroblastoma; SNS, Sympathetic Nervous System; WT, Wilms Tumor.

Our study integrated genomic knowledge in the network-based analysis of RNA-Seq data of six pediatric cancer types to provide a novel biological framework for investigating genes involved in childhood cancers. This comprehensive pan-cancer study relies on the robust definition of gene co-expression modules and their association with particular features of pediatric cancers. The observation of transcriptional profiles and biological functions connect modules to cancer-histotype specific, onco-hematologic and canonical oncogenic processes (Fig. 7). Topological analyses highlight that key regulators of these childhood cancer modules comprise major predisposition genes of pediatric tumors, as well as potential therapeutic targets. The pediatric cancer genes of a tumor type were significantly enriched in the tumor-associated module with strong histotype specificity. Genes targeted by precision therapies are over-represented in a limited number of childhood cancer modules, providing perspectives in the development of precision therapies for children.

As demonstrated for adult cancers, our approach enables investigating cancer genes and shows that multiple cancer types have exclusive hub genes. Adult pan-cancer analyses achieved interesting results in identifying functional gene modules common to cancer, rather than modules specific to tumor types^16^. The present study identifies modules associated with childhood cancers having biological implications in developmental processes. Our findings support the close tie between organogenesis and tumorigenesis in childhood malignancies. The pathogenesis of NBL is tightly related to disruption in noradrenergic neuronal development. The key regulators (*PHOX2B, HAND2*, *PHOX2A*, *GATA2/3*) of this developmental process are also the hub genes of the module found associated with NBL^32,33^. One of its key regulators, *PHOX2B*, is the major predisposition gene to NBL^31^. Therefore, the other central genes of the magenta-NBL module constitute interesting candidates that should be further investigated in the study of the nervous system development and NBL. In support of our findings, *ISL1* has been recently defined as a novel candidate gene for NBL and is also one of the hub gene of the magenta-NBL module^32^. The ALL tumorigenesis is the result of aberrant V(D)J recombinations at the origin of recombinase-mediated deregulated expression of a variety of proto-oncogenes. In the lightcyan-ALL module, the genes are involved in V(D)J recombination processes which is consistent with the physiopathology of ALL and includes, as one of its key regulators, a major predisposition gene for B-cell ALL (*PAX5*). The red-WT module is associated with the ontogeny of the kidney and one of its hub genes *PAX2*, is also believed to be a strong candidate gene for WT and is known as a key player in kidney cell differentiation^34^. Numerous genes co-expressed in the lightgreen-MBL module are related to embryonic brain ontogeny and to key transcriptional programs implicated in MBL pathogenesis^37^. The central regulator *OTX2* of the lightgreen-MBL module is a candidate driver gene for MBL pathogenesis and is responsible for the regulation of cerebellar development and forebrain segregation^27,37^. One of the two modules associated with the AML subtype is related to myeloid-mediated immunity processes. The cancer-histotype specific modules associated with NBL, ALL, WT and MBL are significantly enriched in pediatric cancer genes of the related histotype. Despite revealing modules with functional relevance for the majority of the tumor types, our analysis was not able to pinpoint a module specific to glioma. This is likely the result of the wide heterogeneity of this cancer type characterized by distinct subgroups, as shown in our t-SNE analysis. The hub genes of the cancer-histotype specific modules were enriched in known pediatric cancer genes. Many of these hub genes have still unknown functions or unrevealed implications in childhood cancers. Considering these converging levels of evidence, the hub genes of the cancer-histotype specific modules constitute interesting candidates that should be investigated to validate their role in pediatric cancers, developmental processes, or both.

Our analysis further links modules to cancer-related pathways that are not specific of one pediatric tumor. Statistical analyses show enrichment of cancer genes frequently altered by pathogenic germline variants in the module related to cell cycle regulation and DNA repair, which is consistent with recent findings^2,3^. The genes co-expressed in this module are therefore likely early genetic determinants of childhood tumorigenesis. In acute leukemias, the cancer driver genes are over-represented in the brown module associated with common functions in epigenetic and post-transcriptional modifications. These processes are the most important somatically-altered pathways in childhood cancers and could be critical for tumor progression in hematologic malignancies^3,4^. The ALL driver genes are enriched in the lightcyan-ALL module related to V(D)J recombination and the grey60 module linked to B cell activation and differentiation. This suggests that co-expressed genes and pathways in these modules (lightcyan, grey60) could contribute to B-cell ALL tumorigenesis. We could not assess the genomic alterations for all the studied tumor types because of biases in documented literature. There was a lack of information regarding germline mutations in WT and the driver genes in glioma and MBL that prevented us to test them for enrichment analyses^2,4^.

Regarding over-representation of clinically actionable genes in key modules, our analyses give relevant information about therapeutic targets. Across pediatric malignancies, the canonical oncogenic (tan) module shows a significant enrichment in drug-targetable genes. Most of the central regulators of the tan module are taking part in the regulation of the cell cycle. Currently, number of specific cell cycle inhibitors have emerged in the context of pediatric-focused drug development^48^. Our results thus enable identifying candidate targets in cell-cycle therapeutics in childhood cancer. The majority of the hub genes of the grey60 module have key roles in innate immune recognition and activation and comprise Toll-like receptors (*TLR1* and *TLR6*) that are potential therapeutic targets in onco-hematology^49^. Hematopoietic malignancies promote unique immune evasion pathways and genes taking part in the innate immune system appear to be logical innate immune targets. The hub genes of the grey60 module constitute candidate targets that should be investigated for therapeutics in onco-hematology. Targetable genes involved in the VEGF pathway are enriched in the midnightblue module and include critical regulators such as *VEGFR1* (known as *FLT1*) and *VEGFR3* (known as *FLT4*) that are inhibited by VEGF-targeted approaches (sunitinib, sorafenib, axitinib, pazopanib, cabozantinib, nintedanib, lenvatinib). Further investigations of the genes co-expressed in this module is however needed to clarify their potential in the management of hematologic malignancies.

Pan-cancer analysis of metadata raises several issues related to batch effects that likely contribute to experimental artifacts. In order to prevent such biases, RNA-Seq data available in the TCCI have been processed using the same pipeline of analysis. On these data, we additionally performed a normalization procedure taking into consideration the tumor type and the project associated to tumor samples. We have controlled the relevance of our normalization by checking similarity between TARGET and TREEHOUSE related subsets. As an example, one can note that MBL samples deriving from the TARGET project segregate with brain/nervous system tumor samples from the TREEHOUSE project, rather than the other TARGET samples (Fig. 2). We acknowledge that validating our results by reproducing the framework on comparative external dataset would reinforce the robustness evaluation of the co-expression network. However, many of the consortia that focused on deciphering the genetic etiology of pediatric cancers by generating genomics data are still ongoing. TCCI is the only compendium, to our knowledge, that gathers pediatric pan-cancer transcriptomic data for the six studied histotypes. As no data were available to perform a comparative study, we made a classical robustness validation to evaluate the reliability and stability of the co-expression network. Bootstrap-based methodologies and statistical tests were performed, as done previously in major co-expression studies^13^. Another point is related to interpretability of the modules. As modules can be interconnected, some genes may interact with many others and participate in different functions^50^. This could be seen as a limit, considering that genes involved in various malignancies have lower biological significance than expected, towards a particular tumor. As an example, *WT1* gene is not among the top hub genes of the WT-module because of its involvement in different cancer types. We also questioned the tissue effect in our study, hypothesizing that the modules associated with pediatric tumor histotypes could be more the reflect of the tissue origin of the tumor than independent molecular drivers. We performed additional analyses proving that pediatric cancer genes of one tumor type were over-represented in the tissue-specific genes matching the cell-of-origin of the tumor (Additional File 2: Fig. S3b). This is consistent with the cell-of-origin of a tumor that is likely to retain the embryological molecular networks that are critical to tissue specification and cancer etiology^51^. Our findings support that molecular drivers of the pediatric tumors cannot be considered as independent of the cell-of-origin of a tumor.

## Conclusions

Our integrative approach provides to the clinical and scientific community a detailed characterization of the modules and genes highly associated with main pediatric tumors. Our findings provide a working frame for mechanistic investigations of the biological processes impaired in childhood cancers. Our results constitute a novel resource for cancer-related genes and potential therapeutic targets in childhood malignancies (Additional File 1: Table S2). We provide tumor-specific association metrics for 14,748 protein-coding genes that could constitute novel criteria for future variant prioritization methodologies, while being extended to more childhood tumor types.

## Methods

### Pediatric pan-cancer gene expression data

Pediatric pan-cancer RNA-Seq data were obtained from the Treehouse Childhood Cancer Initiative dataset (released July 2017) and downloaded from the UCSC Xena platform at https://xenabrowser.net/datapages/. RNA gene expression data were available for 11,074 samples and 60,498 transcripts together with associated clinical information (gender, age at diagnosis and tumor type). We selected only cases with an age at diagnosis equal to or less than 18 years old to fit our problematic and cancer types that were represented by at least 50 cases for enough statistical power. The AML, ALL, NBL, and WT samples data were recovered from the TARGET project and supplemented by the MBL and glioma samples from TREEHOUSE. Expected counts were annotated using the human genome (GRCh38.p3) version 23 with Ensembl gene IDs. We focused on transcripts with consistent annotations, i.e. protein-coding genes, with more than 10 reads in overall samples. Read counts were normalized using the variance-stabilizing transformation of the DESeq. 2 R v.16.1 package^52^ based on tumor type and project variables (TARGET, TREEHOUSE). The resulting transcriptome dataset consisted of 14,748 gene expression measurements for 820 pediatric tumor samples, see Extended Experimental Procedures for data pre-processing (Additional File 3).

### Spatial distribution and cluster analysis of pediatric tumor samples

We employed the t-SNE technique to investigate and visualize the transcriptome dataset in a low-dimensional space (2D-map)^53^. To apply t-SNE on more than thousand input objects, we used a variant of the Barnes-Hut algorithm. We ran 2,000 times the Barnes-Hut t-SNE and set the *theta* parameter to 0 to lower the Kullback-Leibler divergence (Rtsne R library v.0.13). The resulting coordinates were used for hierarchical clustering analysis (hclust R stats v.3.4.4) and clusters were defined using the *cutree* function of the R stats library.

### Weighted gene co-expression network analysis

We constructed a co-expression network using the WGCNA method developed by Langfelder and Horvath (WGCNA R library v.1.63)^54,55^. We used the blockwiseModules function to construct a signed co-expression network with sized modules ranging from 30 to 8,000 genes and set the power adjacency function to 14 and the mergeCutHeight to 0.25, see Extended Experimental Procedures for details (Additional File 3). To analyze large dataset with more than 5,000 probes, the function blockwiseModules split automatically the dataset into two blocks. Briefly, we used a pairwise Pearson correlation to calculate a similarity matrix and applied a soft power adjacency function with ß = 14, to best fit the scale-free topology criterion as recommended by the authors. This adjacency matrix represents the connection between gene pairs measured by their similarity of expression levels across pediatric cancer samples. We then constructed a Topological Overlap Matrix (TOM) that was determined by the strength of the shared connection between the gene pairs and their neighbors^54^. In the network like structure, each node represents a gene and each edge between two nodes reflects the connection between genes. The intra-modular connectivity is measured by the sum of the connectivity of one gene with the other genes of one module. The 25% most highly inter-connected genes of one module were defined as the hub genes. A hierarchical dendrogram was constructed based on the TOM matrix and clusters were defined by using a cut height approach implemented in the blockwiseModules function to define modules of genes. The grey module gathered all non-assigned genes and was discarded from statistical analyses. The Module Eigengene (ME) was defined as the first principal component of a given module and considered as a representative of the module expression profile. The Module Membership (MM) of a gene was defined as the correlation between its expression profile and the ME of a module. We tested the difference in the mean expression levels of a gene between one tumor type vs all the other types by performing Wilcoxon Rank-Sum tests (wilcox.test R stats v.3.4.4). P-values were adjusted with a Bonferroni correction according to the number of genes and tumor types tested (*p* = 5.65 × 10^−7^). The gene significance (GS) was measured as minus log10 of the adjusted p-value and reflected the association of gene with a tumor type.

### Robustness of co-expression network construction

To evaluate the stability and reliability of the co-expression network, we assessed if the modules were composed of genes more strongly correlated than by chance^13^. We randomly selected gene sets matching the size of the observed module and compared the sum of gene correlations in the null module with the one observed over 10,000 iterations. The statistical probabilities were defined as the rank of the observed module among null module out of the total iterations. Significance was considered after Bonferroni correction according to the number of modules tested (*p* < 2.17 × 10^−3^). We used a bootstrap-based method to evaluate the module structure vulnerability to perturbations. Networks were reconstructed 100 times with the same parameters on a random selection of the initial samples. The robustness was measured as the number of times a gene was assigned to the observed module over iterations.

### Relationships between pediatric cancers and co-expression modules

We performed a LDA approach (lda function MASS R package v.7.3–50) to segregate pediatric cancer types based on the average expression profiles of each module. The module contribution to the in-between-class variability of a pediatric tumor was defined as the mean expression of the module across samples of one tumor. We obtained a matrix with tumor types in rows and modules in columns with each cell corresponding to this mean expression. To identify strong module-tumor relationships, we defined an empirical threshold of 0.06 of the absolute value of the mean expression. Mean values were visualized through clustered heatmaps (pheatmap R library v.1.0.10).

### Biological pathway characterization of childhood cancer co-expression modules

We performed a functional enrichment analysis using Gene Ontology (GO) and Kyoto Encyclopedia of Genes and Genomes (KEGG) with enrichGO and enrichKEGG functions (clusterProfiler R library v.3.4.4). For each module, the 100 genes with the highest MM were used as input and the initial 14,748 genes as the background set. For the sake of accuracy, we used entrez ids as input and set up the significance thresholds to 0.01 for FDR adjustment method^56^.

### Reference childhood cancer gene, genomic alteration, druggable and tissue-specific gene sets

The pediatric cancer genes (pedCGs) were collated from the PediCan database^29^. Based on the published study of Zhang and colleagues^2^, we selected all the germline variants reported in autosomal dominant and recessive cancer genes to establish pediatric predisposition genes (pedCPGs) for each pediatric tumor. All the alterations in autosomal dominant cancer genes were displayed across modules by using the oncoPrint function (ComplexHeatmap R library v.1.14.0). We used the list of pediatric cancer driver genes (pedCDGs) identified by Ma and colleagues^4^ and selected only the significantly mutated ones for each pediatric tumor type (MutSigCV, *p* < 0.01 or GRIN, *p* < 0.01). Potentially druggable genes (PDGs) consisted of the ones known to have a direct or indirect targeted treatment available or under development^57^. The detailed methodology is available in the Extended Experimental Procedures (Additional File 3). The tissue-specific genes were defined from the GTEx transcriptome data v1.1.9 (https://www.gtexportal.org/home/datasets) of normal tissue samples. The teGeneRetrieval function (TissueEnrich R library v1.5.1)^58^ was used to identify tissue-specific genes based on the median gene-level TPM by tissue.

### Statistical enrichment analysis and visualization

Gene set enrichment analyses were performed using a two-sided Fisher’s Exact test with an alpha level of 0.05 to assess the relationship between the genes of a list and a module. This analysis determines whether the fraction of genes of interest in the module is higher compared to the fraction of genes outside the module (i.e., background set). The statistical probabilities were reported as the FDR adjusted p-values to reduce the likelihood of false positives^58^. All the enrichments with OR >1 passing FDR < 0.05 were considered as significant in the analysis. To visualize relevant genes in the network, we selected the top 15 hub genes and pediatric cancer genes within a module (igraph R library v.1.2). The edges between pairs of the input genes were calculated based on the TOM and represent the strength of their shared connections. The over-representation of pediatric cancer genes in tissue-specific gene sets was displayed using the corrplot function (corrplot R library v0.84).

## Supplementary information

Supplemental Tables.

Supplemental Figures.

Extended Experimental Procedures.

## Data Availability

Publicly available data analyzed in our study were acquired from the Treehouse Childhood Cancer Initiative dataset on the UCSC Xena Platform at https://xenabrowser.net/datapages/ (Treehouse public expression dataset, July 2017).
