# Supplementary material for: Depicting the genetic architecture of pediatric cancers through an integrative gene network approach
Source: Sci Rep. 2020 Jan 27;10:1224. doi: 10.1038/s41598-020-58179-0 (PMC6985191; doi:10.1038/s41598-020-58179-0)
Supplement: Supplementary file 2 — Supplemental Figures. [file 41598_2020_58179_MOESM2_ESM.pdf]

Supplemental Figures

Depicting the genetic architecture of pediatric cancers through an integrative gene network approach

Clara Savary[1], Artem Kim[1], Alexandra Lespagnol[2], Virginie Gandemer[3,4], Isabelle Pellier[5], Charlotte Andrieu[6,7], Gilles Pagès[8,9], Marie-Dominique Galibert[2,3], Yuna Blum[10†], Marie de Tayrac[1,6†*]

[†] Co-contributing author

[*] Corresponding author

[1] Genetics of Development Related Pathologies Team, Univ Rennes, IGDR (Institut de génétique et développement de Rennes) - CNRS UMR 6290, Rennes, France.

[2] Somatic Cancer Genetics Department, Pontchaillou Hospital, CHU de Rennes, Rennes, France.

[3] Gene Expression and Oncology Team, Univ Rennes, IGDR (Institut de génétique et développement de Rennes) - CNRS UMR 6290, Rennes, France.

[4] Department of Pediatric Oncology and Hematology, Pontchaillou Hospital, CHU de Rennes, Rennes, France.

[5] Pediatric Immuno-Hemato-Oncology Unit, Angers University Hospital, Angers, France.

[6] Molecular Genetic and Genomic Department, Pontchaillou Hospital, CHU de Rennes, Rennes, France.

[7] Chemistry Oncogenesis Stress Signaling (COSS) Laboratory – INSERM U1242, Centre de Lutte Contre le Cancer (CLCC) Eugène Marquis, Rennes, France

[8] University of Nice Sophia Antipolis, Centre Antoine Lacassagne, IRCAN (Institute for Research on Cancer and Aging of Nice) - CNRS UMR 7284, INSERM U1081, Nice, France.

[9] Biomedical Department, Centre Scientifique de Monaco, Monaco, Principality of Monaco

[10] Programme Cartes d'Identité des Tumeurs (CIT), Ligue Nationale Contre le Cancer, Paris, France.

(a)

(b)

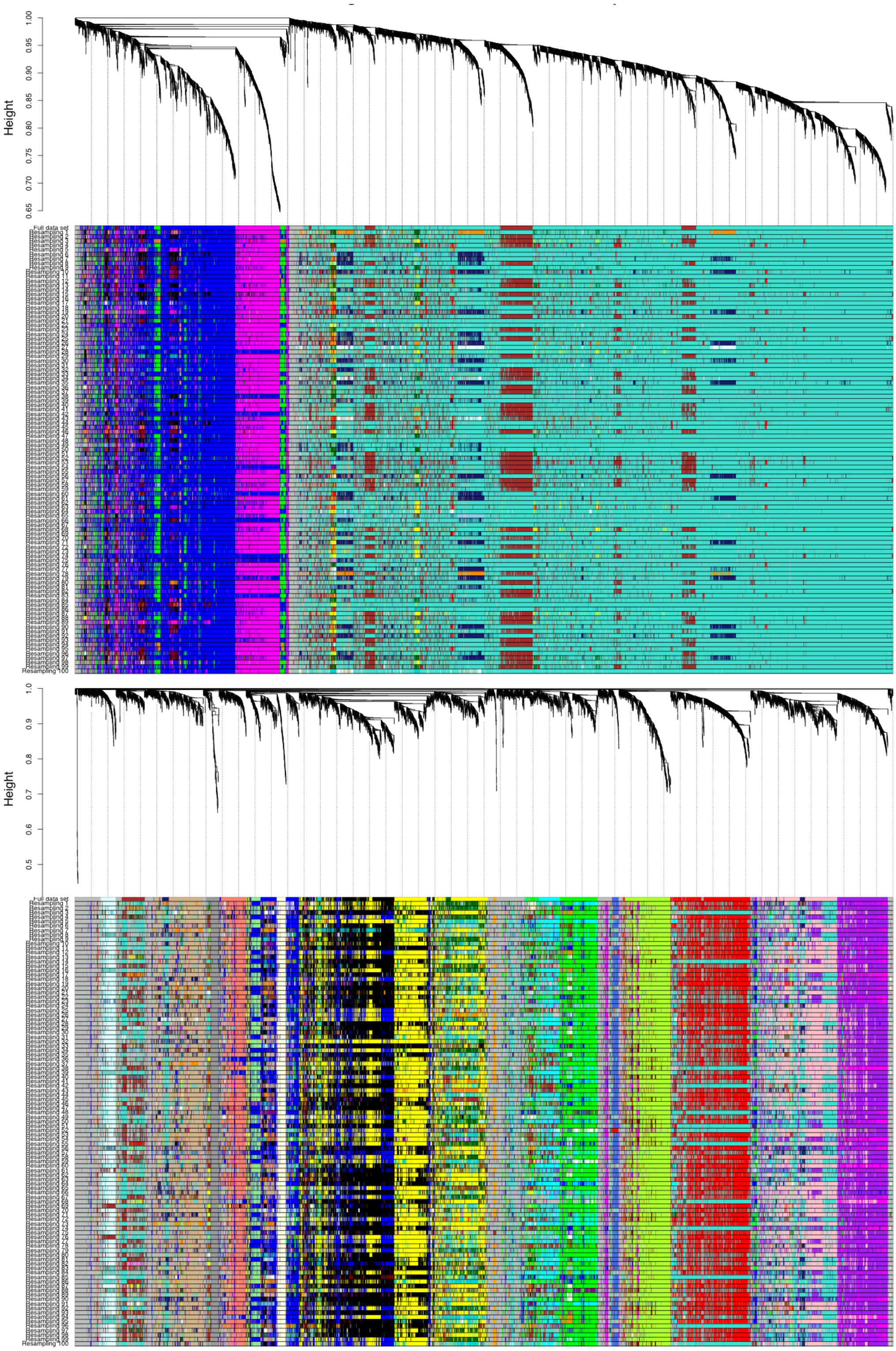

Supplementary Figure S1. Bootstrap analysis of the co-expression modules. Module robustness analysis was performed by reconstructing modules with the same parameters and randomly selecting samples from the initial dataset. Considering its high dimensionality, the dataset has been separated into two blocks of genes (as recommended by WGCNA guidelines). We displayed the module assignment for each gene across the 100 iterations (in rows) in the block 1 (**a**) and 2 (**b**) of genes.

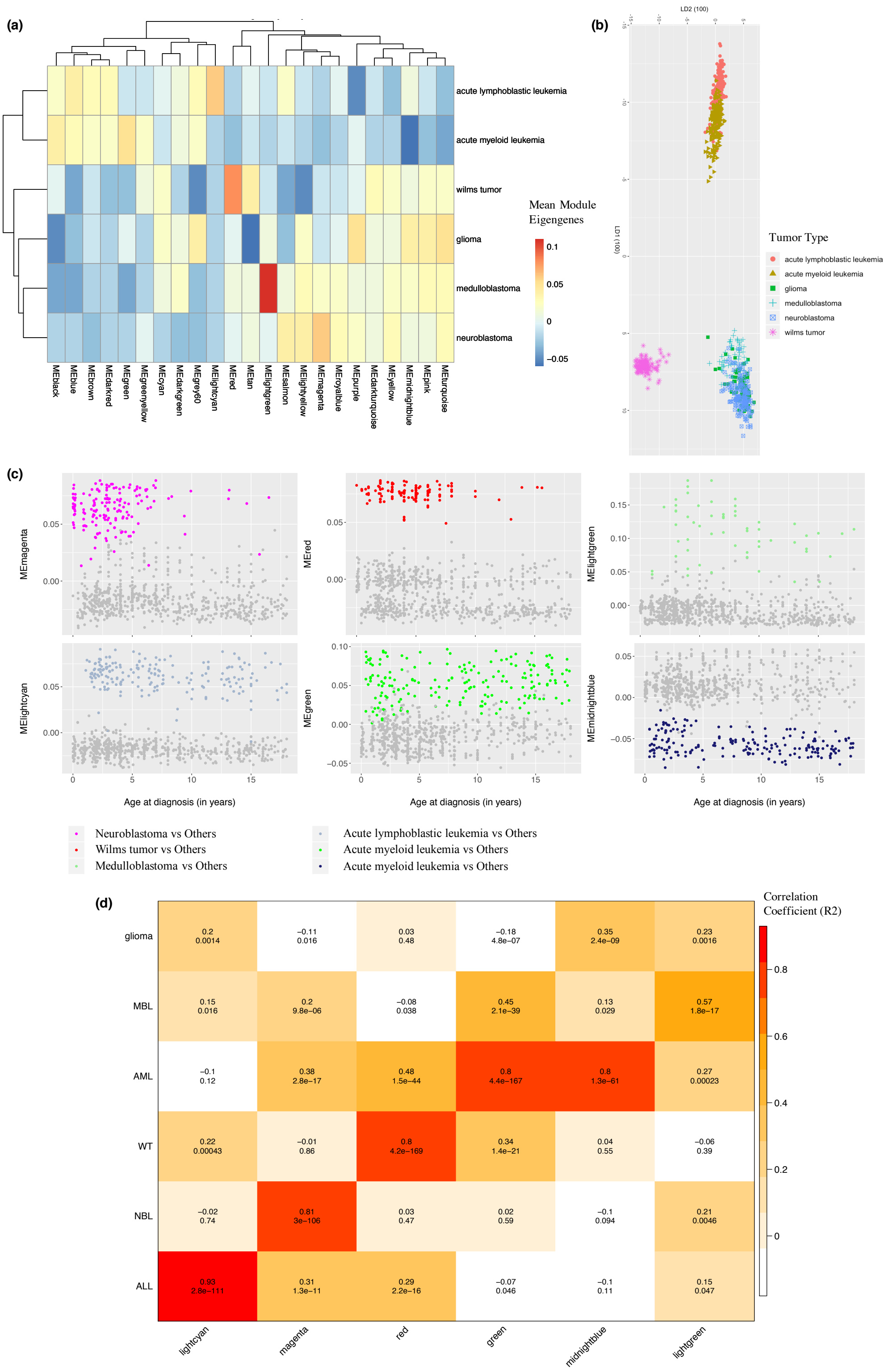

Supplementary Figure S2. Pediatric cancers and co-expression modules relationships.

(a) Heatmap displaying the Linear Discriminant Analysis (LDA) results. Average gene expression profile of modules (Module Eigengenes, ME) (columns) for each pediatric cancer (rows) are displayed in cells and color-coded according to the blue (low values) to red (high values) gradient on the right. **(b)** LDA plot of pediatric tumor samples based on ME values. **(c)** Scatterplot displaying the average gene expression profile of cancer-specific modules across ages at diagnosis in years. Each dot represents the ME value of a pediatric tumor sample. Samples from specific tumor histotype are highlighted according to the module color, while the other tumor types are colored in grey. **(d)** Module-tumor relationships based on the Pearson correlation results between the absolute values of Gene Significance (GS) of tumors (rows) and Module Membership (MM) of modules (columns) are presented. The correlation coefficient R2 (top) and statistical probability (bottom) are displayed in each cell that are color-coded according to the white (low values) to red (high values) gradient (legend on the right). ALL, Acute Lymphoblastic Leukemia; AML, Acute Myeloid Leukemia; MBL, Medulloblastoma; NBL, Neuroblastoma; WT, Wilms Tumor.

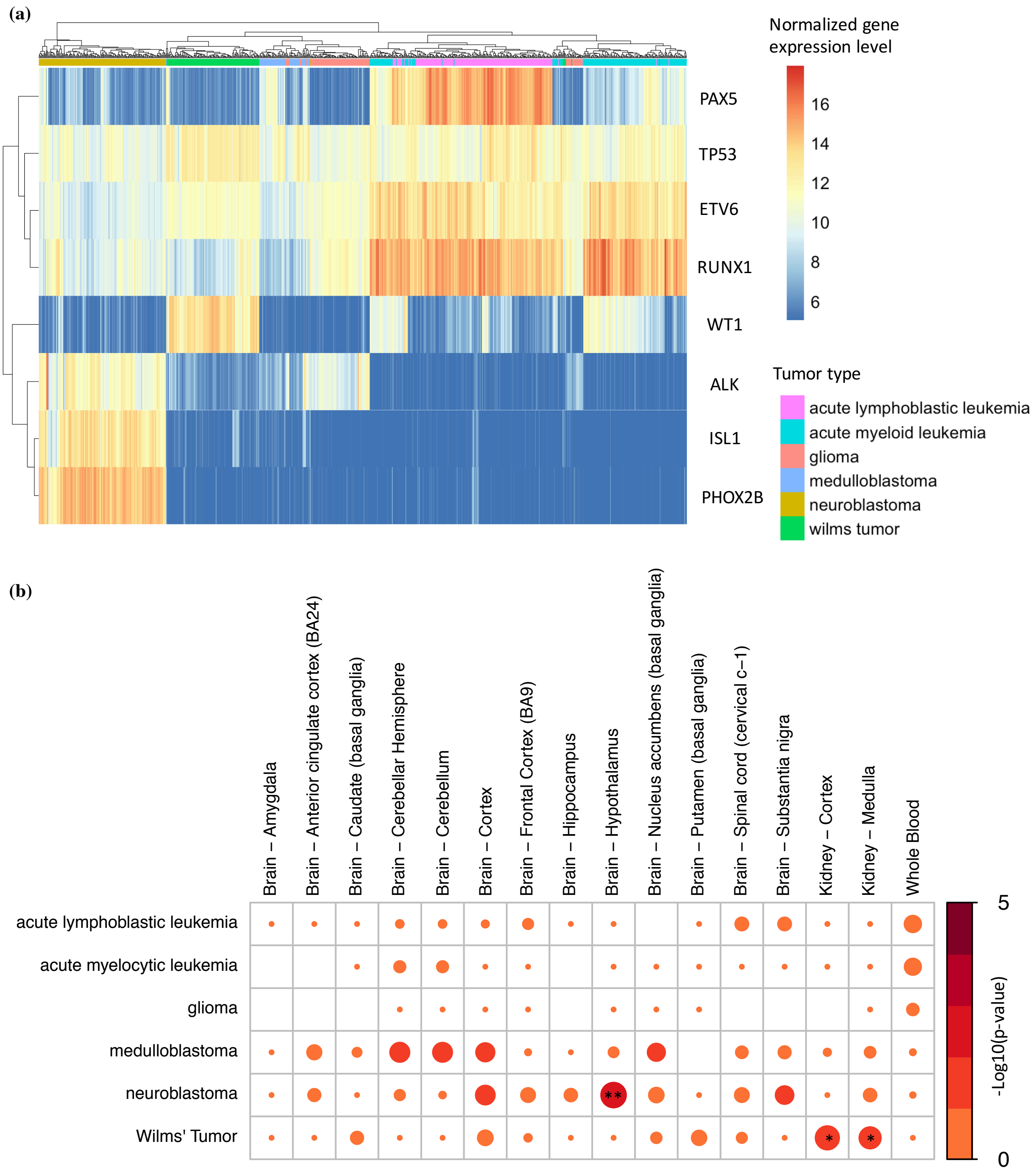

Supplementary Figure S3. Gene expression profiles and tissue-specific enrichment of pediatric cancer genes.

(a) Heatmap showing the normalized expression levels of known pediatric cancer predisposition genes (rows) across tumor samples (columns) with color-coded cells according to the blue (low values) to red (high values) gradient on the right. Samples are labeled by colors according to pediatric tumor histotypes (legend on the right). **(b)** Tissue-level enrichment for pediatric cancer genes (PediCan) of the six pediatric tumors (rows). Enrichment values are displayed as minus log10 of the p-value (*$p < 0.05$, **$p < 0.01$) and results with $p < 0.05$, $OR > 1$ are shown. Pediatric cancer genes for Neuroblastoma are significantly enriched in genes specifically expressed in the Brain – Hypothalamus (OR = 5.43 [1.45-14.3], p = 0.007), and Pediatric cancer genes for Wilms' tumor are enriched in genes specifically expressed in Kidney – Cortex (OR = 6.55 [1.32-19.92], p = 0.012) and Kidney – Medulla (4.95 [1-15.03], p = 0.025).
