# Supplementary material for: Depicting the genetic architecture of pediatric cancers through an integrative gene network approach
Source: Sci Rep. 2020 Jan 27;10:1224. doi: 10.1038/s41598-020-58179-0 (PMC6985191; doi:10.1038/s41598-020-58179-0)
Supplement: Supplementary file 3 — Extended Experimental Procedures. [file 41598_2020_58179_MOESM3_ESM.pdf]

Extended Experimental Procedures

Depicting the genetic architecture of pediatric cancers through an integrative gene network approach

Clara Savary[1], Artem Kim[1], Alexandra Lespagnol[2], Virginie Gandemer[3,4], Isabelle Pellier[5], Charlotte Andrieu[6,7], Gilles Pagès[8,9], Marie-Dominique Galibert[2,3], Yuna Blum[10†], Marie de Tayrac[1,6†*]

[†] Co-contributing author

[*] Corresponding author

[1] Genetics of Development Related Pathologies Team, Univ Rennes, IGDR (Institut de génétique et développement de Rennes) - CNRS UMR 6290, Rennes, France.

[2] Somatic Cancer Genetics Department, Pontchaillou Hospital, CHU de Rennes, Rennes, France.

[3] Gene Expression and Oncology Team, Univ Rennes, IGDR (Institut de génétique et développement de Rennes) - CNRS UMR 6290, Rennes, France.

[4] Department of Pediatric Oncology and Hematology, Pontchaillou Hospital, CHU de Rennes, Rennes, France.

[5] Pediatric Immuno-Hemato-Oncology Unit, Angers University Hospital, Angers, France.

[6] Molecular Genetic and Genomic Department, Pontchaillou Hospital, CHU de Rennes, Rennes, France.

[7] Chemistry Oncogenesis Stress Signaling (COSS) Laboratory – INSERM U1242, Centre de Lutte Contre le Cancer (CLCC) Eugène Marquis, Rennes, France

[8] University of Nice Sophia Antipolis, Centre Antoine Lacassagne, IRCAN (Institute for Research on Cancer and Aging of Nice) - CNRS UMR 7284, INSERM U1081, Nice, France.

[9] Biomedical Department, Centre Scientifique de Monaco, Monaco, Principality of Monaco

[10] Programme Cartes d'Identité des Tumeurs (CIT), Ligue Nationale Contre le Cancer, Paris, France.

Pediatric pan-cancer expression data

The pediatric pan-cancer gene expression dataset was obtained from the Treehouse Childhood Cancer Initiative dataset (released July 2017) and downloaded from the UCSC Xena platform at https://xenabrowser.net/datapages/. This public database is a compendium of the Treehouse partner clinical sites (TREEHOUSE), the Therapeutically Applicable Research to Generate Effective Treatments (TARGET) and The Cancer Genome Atlas (TCGA) databases. RNA gene expression datasets were downloaded for 11,074 samples and 60,498 transcripts together with patient age, gender and disease type. We selected cases with an age at diagnosis less than 18 years old and cancer types represented by at least 50 cases. The acute myeloid leukemia (AML), acute lymphoblastic leukemia (ALL), neuroblastoma (NBL) and Wilms tumor (WT) samples were selected from the TARGET project and supplemented by the medulloblastoma (MBL) and glioma samples from TREEHOUSE. Expected counts were annotated using the human genome (GRCh38.p3) version 23 with Ensembl gene IDs. We focused on transcripts with consistent annotations, i.e. protein-coding genes, with more than 10 reads in overall samples. Read counts were normalized using the variance-stabilizing transformation of the DESeq2 R v.16.1 package[1] based on tumor type and project (1: TREEHOUSE; 2: TARGET) variables. To reduce the noise, we discarded the 25% of the less varying genes and removed one outlier identified by hierarchical

clustering analysis (hclust R stats v.3.4.4. function). The resulting transcriptome dataset consisted in 14,748 gene expression measurements for 820 pediatric tumor samples.

Weighted gene co-expression network analysis

We constructed a co-expression network using the weighted gene co-expression network analysis (WGCNA) method developed by Langfelder and Horvath[2] (WGCNA R library v.1.63). We used a pairwise Pearson correlation to calculate the similarity matrix. We applied a soft power adjacency function with ß = 14, to best fit the scale-free topology criterion as recommended by the authors. The topological overlap matrix was determined by the strength of the shared connection between the gene pairs and their neighbors[3]. Hierarchical clustering was performed on the topological overlap–based dissimilarity matrix with average linkage. The modules were identified by cutting the dendrogram to define stable clusters with a dynamic cut- tree algorithm. The signed co-expression networks were generated using the function blockwiseModules with sized modules ranging from 30 to 8,000 genes and merged when sharing high transcriptional similarities; mergeCutHeight set to 0.25. To analyze large dataset with more than 5,000 probes, the function blockwiseModules split automatically the dataset into several blocks. The Module Eigengene (ME) was defined as the first principal component of a given module and considered as a representative of the module expression profile. Intra- modular connectivity measured the sum connectivity of a given gene to the other genes within the same module and the most highly interconnected genes were defined as hub genes. The Module Membership (MM) was measured by correlating the expression profile of a gene with the ME of a module. The gene significance (GS) reflected the association of gene to external information, e.g. tumor type. The GS evaluated the gene expression differences between tumor groups and was reported as minus log10 of the adjusted p-value of the

Wilcoxon Rank-Sum test (wilcox.test R stats v.3.4.4). P-values were adjusted with a Bonferroni correction according to the number of genes and tumor types tested ($p = 5.65x10^{-7}$).

Reference childhood cancer gene, genomic alteration and druggable gene sets

PediCan (Pediatric Cancer) is the first pediatric gene data resource based on a comprehensive literature curation and data integration[4]. The corresponding dataset (pcdb_gene_735_download.txt) was downloaded from http://pedican.bioinfo-minzhao.org/. For each type of cancer, we used the following keywords: "neuroblastoma", "glioma", "acute lymphoblastic leukemia", "acute myelocytic leukemia", "Wilms' tumor", "medulloblastoma" and collated all the genes matching the queries to constitute a pediatric cancer genes (pedCGs) list for each tumor type. Based on the work of Zhang and colleagues[5], we selected all the germline variants reported in autosomal dominant and recessive cancer genes to establish pediatric predisposition (pedCPGs) in each pediatric cancer histotype. We also displayed all the alterations in autosomal dominant cancer genes across modules by using the oncoPrint function (ComplexHeatmap R library v.1.14.0). We used the list of pediatric cancer driver genes (pedCDGs) provided by Ma and colleagues[6] and selected only the significantly mutated (MutSigCV, $p < 0.01$ or GRIN, $p < 0.01$) for each pediatric tumor type. Potentially druggable genes (PDGs) consisted in the one known to have a direct or indirect targeted treatment available or under development[7].

Supplemental references

1. Love, M. I., Huber, W. & Anders, S. Moderated estimation of fold change and dispersion for RNA-seq data with DESeq2. *Genome Biol.* 15, (2014).
2. Langfelder, P. & Horvath, S. WGCNA: an R package for weighted correlation network analysis. *BMC Bioinformatics* 9, 559 (2008).
3. Zhang, B. & Horvath, S. A General Framework for Weighted Gene Co-Expression Network Analysis. *Stat. Appl. Genet. Mol. Biol.* 4, (2005).
4. Zhao, M., Ma, L., Liu, Y. & Qu, H. Pedican: an online gene resource for pediatric cancers with literature evidence. *Sci. Rep.* 5, (2015).
5. Zhang, J. *et al.* Germline Mutations in Predisposition Genes in Pediatric Cancer. *N. Engl. J. Med.* 373, 2336–2346 (2015).
6. Ma, X. *et al.* Pan-cancer genome and transcriptome analyses of 1,699 paediatric leukaemias and solid tumours. *Nature* 555, 371–376 (2018).
7. Worst, B. C. *et al.* Next-generation personalised medicine for high-risk paediatric cancer patients – The INFORM pilot study. *Eur. J. Cancer* 65, 91–101 (2016).
